# Workforce characteristics of post-conflict disability services in Benghazi, Libya

**DOI:** 10.1186/1472-6963-14-S2-P23

**Published:** 2014-07-07

**Authors:** Rania Hamed, Anne Cusick

**Affiliations:** 1 School of Health and Society, University of Wollongong, Wollongong, NSW, Australia; 2 Australian Health Services Research Institute, University of Wollongong, Wollongong, NSW, Australia

## Background

In 2011 Libya experienced significant unrest. There were many thousands of casualties and increased acquired disabilities. Many rely on local care services.

Health services planning in a post-conflict environment requires accurate workforce information so capacity can be identified and service provision matched. Very little information was available about disability services or the workforce pre-or-post conflict, and what was available was out of date. This study examined workforce characteristics in the largest disability service in Benghazi, the Rehabilitation Handicap Centre.

## Methods

A case study was conducted on site in October 2012. Approval was given by the Research Ethics Committee University of Wollongong, Centre Manager and Libyan Ministry of Health. An investigator developed survey was administered to volunteer employees whose data was anonymously coded.

## Results

It is a single site service, with well-appointed buildings, prosthetic service equipment is extensive and functioning; other rehabilitation equipment is often out-of-order due to maintenance problems. Apart from ancillary staff of 232 employees work across administrative and clinical departments. Outpatient services for community and war-injured exist with waiting lists. Male inpatient services were available; bed-block was a serious problem with scant community-based-rehabilitation and few discharge options.

71 employees responded (n=40 male; 31 female; mean age 39.4 years, SD 8.2, 26 to 66). All Arabic speaking Libyans. Most staff had high-school (17%), secondary school (28%), diploma (14%) or university (19%) education. Participants were therapists (n=20 of 30) administration officers (17 of 135), nurses (n=14 of 67), prosthesis technicians (n=9 of 14), biomedical technicians (n=6 of 11) and administrative managers (n=5; all). There were 2 consultant doctors. Fourteen held supervisory roles in different departments and most were men. About half worked in teams (n=30). Disability workers were paid less than hospital workers; attrition was a problem and pay schedules were disrupted from time to time. Employees worked 6-hours-a-day 5-days-a-week business-hours, except shift-work nurses. Travel to work was by car (66%) or walking (11%) with no public transport and damaged roads. Personal safety en-route was not a problem. Work-roles were: clinical service delivery (46.5%), followed by administration (21.1%) and prosthetic services (11.3%); 37% perceived services had changed post-conflict. The most common diagnoses after the conflict were stroke, complex physical disability and amputation. 32% provided training to patients or families, 13% to the community. Professional development was uncommon: 80% none in the past 12 months, others had a computer course; 72% had none since 2004; 75% reported no employee induction.

## Conclusion

The centre workforce was stable, ageing and needing professional development. Service demand outstripped workforce capacity and infrastructure.

